# Medical and Physical *Intelligence*

**Published:** 1799-07

**Authors:** 


### MEDICAL AND PHYSICAL
### *INTELLIGENCE,*
### (Original and Selected.)

AMONG other literary occurrences deserving the attention of the medical and scientific reader, we take notice of a *Collection of Medical and Physical Works, including the scarcest and most valuable editions, in the Greek, Arabic, and Latin* languages. We are in possession of a catalogue transmitted to us by a foreign litetary friend, who, for upwards of forty years, has spared neither trouble nor expence, to collect this extraordinary assemblage of medical and physical authors, both in manuscript and print: and we may safely assert, that such a number of curious and scarce editions has never before been exhibited.

As the possessor of these works is inclined to dispose of them, if he should meet with a judicious purchaser in this country, we shall only observe, that the greater part consists of works in the most splendid editions, from the invention of the art of printing down to the present time.

Among the *very scarce works*, we shall mention (No. 12 of the catalogue) "*Aristotelis* de coloribus libellus, a *Sim. Portio* latinitate donatus, et comentationibus illustratus gr. et lat. Florent. 1548. 4to." This book is not to be found in any other catalogue. (No. 20.) "*Hippocratis* libri omnes gr. ad vetustos codices summo studio collati et restaurati, per *Jan. Cornarium.* Basil. 1538. fol." The existence of this edition has frequently been doubted by librarians. (No. 621) "Tractatus de morbo fedo et occulto, &c. per *Petrum Pinctor,* Alexandri VI. Medicum. Romæ, 1500. This curious work was condemned and burnt by order of Pope Alexander VI. who, according to the report of his physician had been afflicted with the "morbus occultus:" so that five copies only of this work were saved from the flames.

This valuable collection is equally interesting to the medical philosopher and to the collector of a library. Mr. NEMNICH, the author of the Polyglot Dictionary, offers to furnish any scarce works that may still be wanting to complete the collection; he will enter into immediate negotiation respecting the price of the whole, being at present in London. Catalogues are deposited, and may be inspected at Mr. PHILLIPS's, No. 71, St. Paul's Church-yard; or at Mr. GEISWEILLER's, bookseller, Pall-Mall; who will give further particulars.

---

Mr. NEMNICH, whom we have mentioned in the preceding article, has prepared for the press a work which cannot but prove highly acceptable to every medical reader who is acquainted with the difficulties so frequently occurring in the perusal of foreign works on pathology. We have had an opportunity of inspecting the manuscript of this laborious and useful work, which the author has intitled, "*Nomenclator Pathologicus, Decem Linguis alphabetico ordine digestus.*" The Latin terms are prefixed to the principal part part of the Nomenclature, after which all the names of the diseases hitherto known and described by nosologists, are given in the English, German, Dutch, Danish, Swedish, French, Italian, Spanish, and Portuguese Languages. We make no doubt that the author, who wishes to see this work printed in England, will meet with a respectable and liberal publisher.

---

Among the singular and paradoxical phenomena on the politico-medical horizon of France, we cannot pass over in silence a periodical work published by order of the Minister of the interior; which we fervently wish may be the harbinger of a re-union between the different contending nations. This work is intitled, "*An Account, together with Medical Observations, on a Collection of Memoirs relative to Philanthropic Establishments; translated from the English and German:*" Eleven numbers, from six to eight sheets each. Paris. Agasse.

In this collection of facts, every discovery or hint at improvement is carefully recorded and presented to the general reader, whether it was suggested by English, German, or French philanthropists.

---

Dr. GRUBER, a learned German clergyman, resident in London, has prepared for the press a correct translation of the late Professor *Gren's* * *Elementary Treatise on Chemistry;* an abridgment of his larger work, the second edition of which appeared in 1796.—This eminent philosopher was the partizan of no system, although he has adopted the arrangement of natural bodies, according to the modern principles of the antiphlogistic school: but his works are not designed for theorists only; they abound in facts, in striking illustrations of the phenomena in nature, such as will ever remain valuable and true, even though all theories which have prevailed from the beginning of time should be found defective. In the English edition, the translator proposes to introduce the discoveries and improvements made since the year 1796.

---

In our second Number, p. 198, we quoted an assertion of FOURCROY, who says, in the 85th Number of the "*Annales de Chimie,*' that the works of MAYOW were first brought into general notice, by the edition of them published by Dr. BEDDOES, in 1790.

From the multiplied labours attending a periodical work, and other professional engagements, we could not be expected to make it an object of inquiry, whether Dr. BEDDOES had ever published such an edition. But we think it a duty to repeat here, what Dr. Beddoes has already stated in Mr. NICHOLSON's excellent Journal of Natural Philosophy, No. 28. p. 108. that this is a *wrong assertion,* and that Dr. Beddoes did not republish Mayow in 1790. From the same source we extract the following passages, in answer to M. Fourcroy's exposition of the merits of Mayow.

"In general (says Dr. Beddoes), the French author has bestowed ample praise upon our countryman, but in the estimate of his understanding he has failed most materially. He has neither entered into his views, nor rightly conceived the spirit with which Mayow laboured.

"It would be extraordinary indeed, if a person who outstripped his age, in a degree of which there is no other example in the history of science, had not been endowed with superior comprehension of mind; and Mr. Fourcroy is directly contradicted by Mayow's Dedication.

"Had Mr. Fourcroy known that Mayow died at 27 or 28, he would not perhaps have talked of the thread breaking in his hands, and of his only

---

* See the Account of his Death, and his Writings, in our second Numb. p. 266.

only opening the career. What Mr. Fourcroy advances concerning the caufes of the greater popularity of BOYLE, appears to me ill-founded".

While fome of our European as well as tranfatlantic chemifts, are ftill difputing on the nature and properties of *phlogifton* and *caloric*, Profeffor WERNER, of Gieffen, in his work, "*On Ætiology*", and "*The Journal for propagating Truth*", has lately endeavoured to prove that, according to his peculiar philofophic fyftem, and from an analytical inquiry into the nature of our fenfes, *there exifts neither matter of heat*, *caloric*, *nor matter of light*, *lumiere;* although our naturalifts and philofophers have beftowed much time and great attention, on thefe elementary fubftances.

Mr. NOTCUTT has fucceeded in obtaining a globule of glafs from the *equifetum hyemale*, or Dutch rufh, ufed in manufactories for polifhing glafs, &c. Mr. H. DAVY, of Clifton, has examined this plant, and found that its epidermis is almoft wholly compofed of filex, which is reticularly difpofed, like that of the bonnet-cane, or calamus rotang. '*Nicholfon's Journal*,' No. xxviii. p. 138.

In the 88th number of the '*Annales de Chimie*,' the ingenious VAUQUELIN has inferted a paper '*On the Excrement of Fowls, compared with their Nourifhment;* with fome Obfervations on the Formation of the Egg.' He found in the afhes of the oat-grain, filex and phofphat of lime. Mr. DAVY obferves on this occafion, that he has not yet had time to examine any grains or feeds, but it is more than probable that the filex exifts in the epidermis of the oat-grain, and not in the farina. The hufks of wheat and barley, as well as the feeds of grapes, and hard fhells, and capfules of fruit, will, he has no doubt, will be found to contain flint. *Ibid.*

At a late meeting of the Royal Academy of Sciences, at Berlin, M. ACHARD read an account of fome curious experiments, made with a view to determine the influence of the compreffion of air on the *vegetation of grain*, and its action on animal life; together with a defcription of a new mode of injecting the capillary tubes of plants.

In confequence of the former experiments made by Profeffor LOWITZ, M. DEVAUX, of Peterfburgh, communicates the following fimple *procefs for freeing maloffes from their fharp tafte*: To 24lb. of molaffes, let 24lb. of pure water be added, together with 6lb. of charcoal in coarfe powder, and let the whole be boiled for half an hour over a moderate fire. This mixture is then to be poured into a fhallow veffel, where the charcoal will fubfide to the bottom: and, after the liquid has been poured off, it is again expofed to the action of heat, fo that the fuperfluous water may evaporate and the molaffes be reduced to its former confiftence. By this procefs the molaffes becomes much milder, and is rendered fit for various culinary ufes.

ProfefforTROMMSDORFF, in a letter to VAN MONS, obferves, that one of his friends has difcovered *calcareous earth in a cryftallized ftate*, in the form of fine needles. This difcovery the Profeffor confiders as a further fupport of his late propofal, for claffing that earth among alkaline bodies.—"Annales de Chimie".—No. 86. p. 221.

M. KLIPSTEIN, one of Trommfdorff's pupils, has lately analyfed a fpecies of *quartz*, of a violet colour, found at Obenwalde, in Germany: it contained 0.40 of filex, 0.53 of alumine, 0.03 of magnefia, and 0.05 of oxyd of iron.—*Ibid.* p. 222.

In

In combining, by digeftion, ether with the red oxyd of iron, TROMMSDORFF obferved, that the oxyd was diffolved, and a *martial ether* readily obtained. This is fhorter and more certain than the former method (of diffolving the iron filings firft in fulphuric acid, evaporating the folution, then re-diffolving the cryftals *per deliquium*, and afterwards mixing the fluid with equal proportions of alcohol and vitriolic ether), and confequently deferves to be adopted in the practice of pharmacy.—*Ibid.*

The fame Profeffor has again made a great number of experiments, with a view to reduce *zircone*, but they have all been unfuccefsful; and he is confequently of opinion that this fubftance ought to retain its place among the earths. The pruffiat of potafs, contrary to the fuppofition of the celebrated VAUQUELIN, does not precipitate *zircone*, though in a pure ftate. *Ibid.* p. 223.

The *zoonic acid* will probably lofe its former particular rank affigned to it by the claffification of *Berthollet* (namely that of partaking both of animal and vegetable nature). *Trommfdorff* fays, that he has much reafon to think it is analogous to the febacic acid, which formerly engaged the attention of Von Crell. *Ibid.*

WIEGLEB has chemically analyzed the *black chalk of Bayreuth*, and found that this mineral does not effervefce with acids; when expofed to the action of fire it gradually lofes its black colour, affumes a reddifh appearance, and at length experiences a deficiency of 185. Its component parts are as follow: filex 64.5; alumine 11.25; ferruginous earth 2.75; carbon 11; concrete water (hydrogen) 7.5. Total 97.00. Lofs 3.—*Ibid.* No. 88. p. 13.

VAN MONS has furnifhed us with a method of *purifying gum refins*, which deferves the attention of both the chemift and the apothecary. He firft propofes to treat them by way of emulfion and trituration in a mortar; to thofe gums which contain an excefs of refin, he adds a fmall quantity of gum arabic, or that of the cherry tree, to re-unite the refinous milky liquor, and he then evaporates them to a proper confiftence, by means of the water bath. *Ibid.* No. 29. p. 210.

M. HILDEBRANDT has contrived a new method of preparing *the red oxyd of mercury*, by means of the nitric acid. He firft directs to deprive the nitrat of mercury of its water of cryftallization, to reduce it to powder, by calcining it in an earthen crucible, while it muft be fhaken during the whole procefs. The French editor, on this occafion, obferves that the oxyd thus obtained can never have a fine red colour, but muft be of a reddifh brown and earthy appearance. *Ibid.* p. 212.

KASTELEYN informs us of a *new method of making vinegar.* A mixture, confifting of 72 parts of water, and 4 parts of a rectified fpirit of grain, being expofed to a proper temperature, for the fpace of two months; is during that time converted into perfect vinegar. The editor of the "Annales de Chimie' remarks, that this procefs, which is the invention of M. Heber, of Berlin, is not always fuccefsful. *Ibid.* p. 214.

Dr. WILLICH has in the prefs a fecond edition of his '*Lectures on Diet and Regimen*,' with confiderable improvements and additions, which will appear early in Auguft.

